# What is the Journey to Physiotherapy Care for Women over 60 Years with Pelvic Floor Dysfunction: Scoping Review

**DOI:** 10.1007/s00192-026-06544-4

**Published:** 2026-02-25

**Authors:** Laura Murphy, Gemma Morcombe, Divya Subramaniam, Madeleine Walker, Rob Waller, Michelle Franklin, Marissa Joseph, Amy Tinetti

**Affiliations:** Present Address: Curtin University - Curtin School of Allied Health, GPO Box U1987, Perth, WA 6845 Australia

**Keywords:** Female, Elderly, Pelvic floor disorder, Physical therapy, Referral

## Abstract

**Background:**

Pelvic floor dysfunction (PFD) impacts 20% of adult women with prevalence increasing with age. Physiotherapy is considered first-line care and can greatly improve quality of life for those experiencing symptoms. However, few women over 60 seek professional help. Little is known regarding how this population receives care for their PFD, including if, and when, they are referred to physiotherapy. The aim of this study is to review the current literature on the journey of women over 60 years with PFD to accessing physiotherapy.

**Methods:**

A search of electronic databases was completed to inform the scoping review. Articles were included if they were in English and analysed the journey to physiotherapy for women 60 years or over with PFD. Analysis of the studies were completed by reviewers and organised into key themes.

**Results:**

Four articles met inclusion criteria and were included in the scoping review. Four themes were identified: the General Practitioner (GP) as gatekeepers to pelvic care, timely initiation of physiotherapy care, bias towards passive care for women over 60 with PFD and lack of awareness around the benefits of physiotherapy for PFD in women over 60.

**Conclusions:**

Given the limited literature found on this topic it is difficult to draw conclusions about the current pathway to physiotherapy for women over 60 years with PFD. However, the available literature suggests this population is less likely to be referred to physiotherapy and more likely to be prescribed passive care, which differs from international guideline recommendations and younger age groups.

**Supplementary Information:**

The online version contains supplementary material available at https://doi.org/10.1007/s00192-026-06544-4.

## Introduction

Pelvic floor dysfunction (PFD) affects 20% of adult women, with the prevalence and advancement of condition increasing with age [1, 2]. PFD refers to a collection of symptoms and anatomical changes associated with suboptimal functioning of the pelvic floor [3]. PFD is a broad term used to encompass bladder, bowel and sexual dysfunction, pelvic organ prolapse (POP) and pelvic pain excluding isolated joint related pain [4]. Older women have a higher prevalence of PFD due to accumulation of several factors influencing PFD, such as childbearing, menopause and comorbidities [2, 5]. It is predicted that by 2030, the number of community dwelling women over 60 years of age in Australia with urinary incontinence (UI) and faecal incontinence (FI) will be 1,405,457 and 363,272 respectively [6].

Pelvic floor dysfunction in women over 60 years has a significant individual, societal and economic impact. This can adversely affect their physical, emotional, and sexual wellbeing, and overall quality of life [5, 7, 8]. Incontinence is a risk factor in older women for admission to residential aged care (RAC) and hospital, increased length of hospital stays and a higher mortality rate [9–11]. The number of Australians in 2030 living in RAC with UI and/or FI is predicted to be 253,113 [6].

The Australian estimated incontinence-related healthcare cost for 2023 was $2.6 billion, with $505.8 million estimated for RAC and formal care (i.e. paid professional care) [5]. An additional $705.8 million was estimated towards the cost of aids and equipment to manage incontinence [6]. Previous studies highlighted the importance of timely referral to pelvic care to reduce the overall associated burden of PFD [7]. Physiotherapy is recommended as part of a multidisciplinary team (MDT) approach for PFD care [12]. According to international guidelines, those who suffer with UI and/or POP can see improvements within 3–6 months from physiotherapy care [13]. The known benefits of physiotherapy include reducing PFD risk factors through education and lifestyle changes, optimising PFM awareness and function, and ultimately reducing the need for surgical intervention [12–14]. Despite international guidelines recommending physiotherapy as part of first-line care for PFD, it is often underutilised by women over 60 [12–15].

A cross-sectional study by Tinetti [15] found that only a limited number of women over the age of 55 with PFD seek professional help. The main drivers for help-seeking behaviour were found to be the level of symptom bother, progression of symptoms and learning about available treatments [15]. This evidence highlights the importance of the right care at the right time [15].

Currently, it is unknown how women over 60 years access physiotherapy care. Opportunity exists to improve education and pelvic care access for women over 60 years if we understand where and how they first seek help for PFD.

### Aims

The primary aim was to (1) review the current literature on the journey of women over 60 years with PFD in accessing physiotherapy care, including referral pathways. The secondary aim (2) was to inform how this population can access and gain benefits of physiotherapy care for PFD.

## Methods

### Protocol and Registration

This scoping review was completed in accordance with Preferred Reporting Items for Systematic reviews and Meta-analysis Extension for Scoping reviews (PRISMA-ScR) [16]. Ethics approval was not sought as it is a scoping review of publicly available data. The proposal was registered with Open Science Framework June 18, 2024 (https://doi.org/10.17605/OSF.IO/FPTY2).

### Eligibility Criteria

#### Population

The inclusion criteria for this study were publications reporting on the journey of women over 60 years with PFD to physiotherapy. This includes bladder, bowel and sexual dysfunction, POP, and pelvic pain. The age 60 was chosen to ensure women would be in the postmenopausal period. Studies involving women of all ages were included when their data could be disaggregated by age. For this review, when the term woman or women is used, this refers to people assigned female at birth. This study excluded pelvic pain conditions that involve the sacroiliac joint, pubic symphysis, hip joint, and lumbosacral regions in the absence of PFD.

#### Concept

For inclusion in this scoping review, publications must have considered the journey of women to physiotherapy care. This could have been via General Practitioner (GP), specialist, self-referral, word of mouth or other methods.

#### Context

Physiotherapy care in all settings was considered. This included private, public, hospital, multidisciplinary and community settings. All studies regardless of geographical location, culture, ethnicity, socioeconomic background and written in English were considered. Guidelines and government websites were screened for eligibility when identified in the search.

### Search Strategy

A preliminary search was conducted to determine if there were any current reviews on this research topic. A librarian was consulted in refining the preliminary search strategy. This search was conducted through ProQuest, CINAHL and Scopus. No scoping or systematic reviews were found. Terms were selected for the full search strategy, demonstrated in Table 1.

The search was conducted by two independent reviewers (GM and MW) between June 23 to August 13, 2024. The search strategy was modified for each database and information source. The search was completed on ProQuest, Web of Science, PubMed, CINAHL, Scopus, Pedro, Google Scholar, Google, DuckDuckGo, Trove and Australian Open Access Repositories. Supplementary File 1 details the full search. All study designs were considered if they were published in English. Reference lists from these articles were put into Scopus to identify if further studies met the criteria.

To limit Google biasing searches based on individual accounts, Google searching involved reviewers signing out of all personal accounts. Researchers also cleared their history and cookies prior to beginning each search. Grey literature from Google and DuckDuckGo were limited to the first 100 hits.

### Selection of Sources of Evidence

**Fig. 1:**
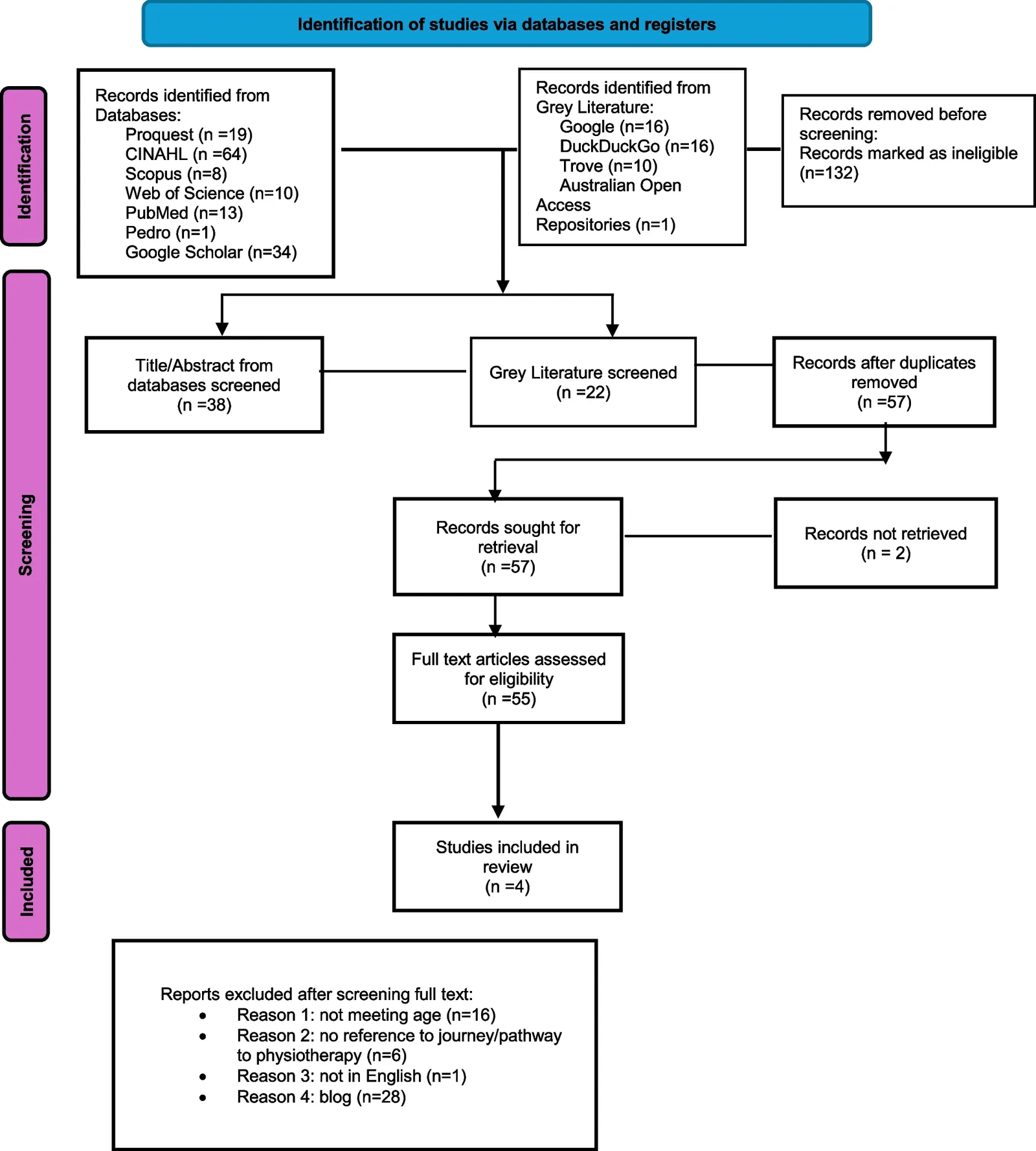
Study procedure flow chart

Titles and abstracts were screened by two independent reviewers (GM and MW) against the inclusion and exclusion criteria, listed in Table 2. The articles that met the criteria by commenting on the journey of women over 60 years to physiotherapy were entered into EndNote and duplicates removed. The same two independent reviewers assessed full texts of selected sources against the inclusion criteria. Reasons for exclusion of sources were recorded. Grey literature that met the inclusion criteria included referral guidelines for health professionals and government websites. At each stage, disagreements were discussed and resolved by a third reviewer (DS). The results of the search process are presented in a PRISMA-ScR flow chart, Fig. 1 [16].

### Data Charting Process

Two independent reviewers (GM and MW) extracted relevant information from the studies into a data collection table, see Table 3. The characteristics extracted included authors, year, country, study design, purpose, number of participants, type of PFD, data collected and the journey to physiotherapy care.

### Synthesis of Results

Analysis of the studies was completed by the reviewing team (GM, LM, DS, MW). Discussion and interpretation followed the analysis. Tables were used to organise key themes, identify similarities and differences between articles, and comment on referral pathways.

### Amendments to Registration Protocol

After registering the protocol with Open Science Framework, and while conducting this scoping review, small amendments to the methods were made. As mentioned in the search strategy, the database DuckDuckGo was searched for grey literature. DuckDuckGo was utilised as it does not track searches therefore it would have less influence over the search. Few sources met the inclusion criteria so the original date of search between 2014 to 2024 was removed, and no limit was set.

## Results

### Selection of Sources of Evidence

The database and grey literature search resulted in 192 sources of evidence. After reviewing titles and abstracts, 60 sources remained. Blogs were excluded as these did not comment specifically on the journey of women or were not specific to age. Following the removal of duplicates, 57 full texts were reviewed. Two full text articles were unable to be accessed; 55 full text articles were assessed for eligibility. Four studies were identified for data extraction. This may have resulted in relevant literature being missed. Figure 1 details the results of the selection process.

### Characteristics of Sources of Evidence

Table 3 details the characteristics of the included studies. The four eligible studies were from the United Kingdom, Australia, the United States and the Netherlands. All four studies included adult women with UI and other combinations of PFD. There were 5529 women analysed in the four studies. Three studies allowed disaggregation of results to below and above 60 years of age [17–19]. The fourth study showed the mean age of 59.8 (SD 14.5) years for the whole cohort [20]. This study split groups into menopausal status, of which 68% were post-menopausal, with the mean age not reported. As our interest is PFD in post-menopausal women, this study remained included in the scoping review. All studies reported quantitative data and two included qualitative data from questionnaires completed by physiotherapists and patients. Included studies were published between 2009 and 2024.

### Synthesis of Results

The synthesis of results identified four key themes. These were (I) GPs as gatekeepers to pelvic care, (II) timely initiation of physiotherapy care, (III) a bias towards passive care for women over 60 years with PFD and (IV) a lack of awareness around the benefits of physiotherapy in women with PFD.

### Theme I: GPs as Gatekeepers to Pelvic Care

All four studies reported the GP as a key journey start point for women’s pelvic care [17–19]. Howard [20] examined women who were managed in a physiotherapy led pelvic health clinic (PLPHC), after initial GP referral. Leubke [18] and Van Gerwen [19] examined referrals to physiotherapy and specialists, after being seen by the GP. The ACPWH [17] study assessed patient self-referral to physiotherapy. This consisted of 15.5% self-referring versus the GP referrals of 66.6% [17]. The percentage breakdown of referrals was not disaggregated by age [17].

### Theme II: Timely Initiation of Physiotherapy Care

All four studies considered either wait lists or time to physiotherapy care for women over 60 years. Van Gerwan [19] reported that women over 60 years are referred less frequently than younger women. In the first 3 months after diagnosis of UI, 12.9% of women 60–74 years and 6.5% aged ≥ 75 years were referred to a specialist or physiotherapist [19]. Van Gerwan [19] identified 29.2% of women 29–59 years were referred to physiotherapy care.

The ACPWH [17] study reported a tendency for older patients not to self-refer. From the data available in this report that was disaggregated by age, the middle age groups (45–64) had a higher proportion of self-referrals (reported via bar graph) before it consecutively reduced each 5-year age bracket [17]. Patients that completed the online survey perceived several benefits of self-referral; easier access to services, saved time and reduced waiting times compared to other referral options [17]. Of the participants who completed an online questionnaire, 87% either agree or strongly agreed that self-referrals could save a lot of time [17].

Luekbe [18] examined electronic health records (*n* = 4382) on time to receive guideline-based care for women aged 18 to 89 years. This audit found patients presenting with stress and mixed UI were more likely to be referred to physiotherapy compared to patients with urgency UI [18]. Only 34.3% of women with a new diagnosis of UI received guideline-based referrals or medications within the first year [18]. For all women with a new UI diagnosis, 3.2% were referred for pelvic floor physical therapy and only 57.4% of those completed their first referral appointment [18]. Age did not significantly change the likelihood of referral for pelvic floor physical therapy.

Howard [20] reported their PLPHC reduced wait times to care when compared to a urogynaecology clinic. The mean wait time for patients to see a urogynaecologist was 80 days, compared to a mean of 16 days on the PLPHC waitlist [20].

### Theme III: Bias Towards Passive Care for Women Over 60 Years with PFD

Two of the four studies in this review reported women over 60 years were more likely to receive passive care for PFD when compared to women 25–59 years [18, 19]. Van Gerwan [19] found that GPs provide prescriptions for incontinence pads and medications at a higher rate for over 60-year-olds compared to younger women. The likelihood of being prescribed incontinence pads was 18.7% (OR 0.8 [95% CI 0.3–1.8]) for women 60–74 years, compared with 19.5% for 75 years and older [19]. Comparatively, only 5.4% (OR 0.2 [95% CI 0.1–0.5]) of women aged 25–59 were prescribed incontinence pads, which is a significant likelihood when compared to those over 60 years [19]. The likelihood of being prescribed medications was 14.4% (OR 0.6 [95% CI 0.3–1.3]) for 60–74-year-olds and 19.5% for the comparison group of 75 years and older [19]. Comparatively, women 25–59 years had a significantly lower likelihood of being prescribed medication (7.8%, OR 0.3 [95% CI 0.2–0.7]) [19].

Luebke [18] reported on the likelihood of medication prescription for women with urinary urgency and mixed UI the year after diagnosis. The 70–79-year-olds were no more likely to be prescribed medications when compared to 60–69-year-olds (OR 1.19 [95% CI 0.90–1.58]) [18]. Yet 80–89-year-olds were significantly more likely to be prescribed medications when compared to 60–69-year-olds (OR 1.39 [95% CI 1.01–1.92]) [18].

### Theme IV: Lack of Awareness Around Benefits of Physiotherapy for PFD in Women Over 60 Years

The two studies that included qualitative data, commented that women were lacking knowledge of physiotherapy care for PFD [17, 20]. This is highlighted in quotes from ACPWH [17].

“I would have taken action a lot sooner if I could have made direct contact with the physio department. When first noticing I had a problem I delayed making an appointment with the GP as I felt it might be too trivial a problem when others might be in need of an appointment. Wider education of what physios can offer would be needed for self-referral to be a first route of choice” [17 (p 29)].

“I never knew of the women’s health (physiotherapy) department before I had my problem, but I now know that they do invaluable good work. Thank you.” [17 (p 29)].

Despite the lack of knowledge around physiotherapy care, Howard [20] found that 98% were satisfied seeing a physiotherapist first at an initial appointment. After receiving care in the PLPHC, 70 participants completed a satisfaction questionnaire and 100% reported that they were satisfied with the knowledge of the physiotherapist [20].

## Discussion

To our knowledge, this scoping review is the first to examine literature reporting the journey to physiotherapy for women over 60 years with PFD. It is difficult to draw conclusions due to the lack of evidence found in this scoping review. The results imply that women over 60 years with PFD predominantly utilise GPs as their first access point to care. GP referral rates to physiotherapy are reduced in older women when compared to younger age groups. This scoping review found that older women were more likely to receive passive care for PFD, compared to younger women. This differs from recommendations in international guidelines [12–14].

All four studies indicated that GPs are the key gatekeepers to primary healthcare for this population, across a variety of health settings in four high-income countries. Previous studies support GPs as the most accessed primary health professional for complaints of PFD [15, 21]. The common theme of GPs as gatekeepers to primary healthcare suggests the need for further studies investigating GP beliefs and knowledge of indications for appropriate guideline-based care for PFD within this age group. These findings highlight that GPs appear to be the best place to allocate resources and raise awareness of the benefits of physiotherapy for women over 60 years with PFD.

All studies commented on wait times to pelvic care, although not all were specific to time to physiotherapy care as referrals were not always progressed [17–20]. The Australian study highlighted engagement with physiotherapy, via the PLPHC, was associated with timely initiation of care [20]. The efficient initiation of care was attributed to the co-location of the PLPHC and urogynaecology outpatient department, which also strengthened interprofessional communication [20]. These findings indicate that older women with PFD who receive physiotherapy care that is collocated in a multidisciplinary clinic are highly satisfied, are managed effectively and in a timely manner [20]. The MDT approach with specialised skills is reinforced as appropriate high-value first-line care for PFD and demonstrates benefits of having co-located multidisciplinary pelvic care. More research is required to examine GP referral rates and the professional collaboration of GPs and physiotherapists specifically for PFD. This may serve as important for helping to improve pelvic care and reducing the total burden of PFD for this age group.

Two studies highlighted GPs and primary care medical practitioners bias towards passive care for older women with PFD compared to younger age groups, such as medication prescription and incontinence pads [18, 19]. These findings allude to GPs and primary care medical practitioners not prioritising active care, such as physiotherapy. Additional research is required within this area to assess whether there is a bias towards passive care for women over 60 years. Specific exploration into why GPs do or do not refer to physiotherapy should be investigated for this population.

A lack of patient awareness of physiotherapy and its benefits for management of PFD was identified [17, 20]. This theme was not specific to the older age group and was commented on generally, and therefore illustrates a gap in the literature for women over 60 years and patient awareness of physiotherapy and its benefits to manage PFD.

### Implications for Research and Clinical Practice

Further research is required to better understand the journey to physiotherapy care for women over 60 years. Review of the current service delivery model of care for PFD and investigating if this model assists women over 60 years to receive the right pelvic care the first time is recommended.

It is known that GPs have limited time and are triage systems for patients [22]. These factors could influence their ability to follow guidelines and address all health concerns in the older age group, who often present with multiple comorbidities. PFD in this population is associated with a deterioration in physical function, frailty and falls, further burdening healthcare systems [9, 10]. The impact that PFD has on healthcare resources will grow with the aging population and the increased mean life expectancy. It is imperative a more multidisciplinary and active approach is sought when addressing PFD in women over 60 years.

The introduction and rise in social media create opportunities for better access to information on appropriate pelvic care and where to seek help. Despite an increasing trend of older Australians utilising the internet, attitudes have not significantly changed [23]. The majority of older Australians report that the internet is challenging to use [23]. ACMA [23] reported that 22% of older Australians (65 + years) access one social media platform and are less likely to engage with social media for content purposes. An identifiable gap exists for older women on the best way to educate and disseminate evidence-based pelvic care.

Future research should be directed at models of care delivery, monitoring rates and referral patterns from primary healthcare providers to physiotherapy for women over 60. Further investigation is needed into the beliefs and attitudes of older patients towards physiotherapy for PFD. Like the survey-based study completed in the UK for UI, it would be valuable to develop survey-based studies specific to this population in other countries [24].

There would be benefit in understanding the prevalence and impact of PFD in older women in low-middle-income countries, remote locations and varied cultures. Low-middle-income countries and those living remotely have restricted access to expert care. The development of educational materials is needed to help address this issue [25].

### Strengths and Limitations

The strengths of this scoping review include the pragmatic design and adherence to the PRISMA-ScR [16]. This has resulted in a comprehensive, reliable and reproducible review of current literature on the journey to physiotherapy for women over 60 years with PFD. The studies found had diverse geographical context (the United Kingdom, Australia, the United States and the Netherlands), which provides an interesting comparison of high-income countries’ approaches to current care involving physiotherapy in this population.

The variability of health systems and paucity of relevant studies found indicate the results are to be interpreted with caution. This includes potential varied referral practices for different countries, associated costs in respect to access to healthcare, including physiotherapy, physiotherapists as first contact practitioners compared to second contact, the role and time constraints of GPs and varying cultural beliefs. Each variability may impact the referral pathway for women over 60 years in a different way. It was difficult to draw conclusions on the overall journey of women to physiotherapy due to the limited literature and evidence found.

## Conclusion

Given the limited literature found on this topic it is difficult to draw conclusions about the current pathway to physiotherapy for women over 60 years with PFD. However, the available literature suggests that this population are less likely to be referred to physiotherapy and are more likely to be prescribed passive care, which differs from international guideline recommendations and younger age groups. Given the impact of PFD in older women, there is a need for further research to inform better care and educational resources.

## Electronic supplementary material

Below is the link to the electronic supplementary material.

Supplementary file1 (PDF 161 KB)

## Data Availability

The data that support this study are available in the article and accompanying online supplementary material.

## Appendix

Tables 1, 2, 3 and Fig. 1

**Table 1:** Search strategy terms

	Participants	Concept	Context
	Women over 60 years	Pelvic floor dysfunction	The journey to physiotherapy care
Search terms	Female over 60 years Women over 60 Women Female Older age Post-menopausal Menopause Older population Aging women	Incontinence Urinary incontinence Fecal incontinence Anal incontinence Bladder dysfunction Bowel dysfunction Sexual dysfunction Pelvic organ prolapse Dyspareunia Vulvodynia Pelvic pain Pelvic floor disorder Pelvic floor disease	Pelvic health physiotherapy Pelvic health physical therapy Physiotherapy Physical therapy Journey Referral Pathway

Table 2

**Table 2:** Inclusion criteria

Inclusion criteria	Exclusion criteria
Women aged 60 years and over Pelvic floor dysfunction Journey to physiotherapy care All care settings All study designs Government websites Clinical guidelines	Studies where results were not able to be disaggregated by age Non-English articles Full text not available Blogs Pelvic pain from musculoskeletal origin in the absence of PFD

Fig. 1

**Table 3:** Characteristics of sources of evidence

Author(s)	Country	Study design	Purpose	Number of participants	Type of PFD	Data collected	Journey to physiotherapy care
ACPWH, 2013	United Kingdom	Report	To evaluate the impact of introducing self-referral to women’s health physiotherapy, evaluate the impact of widening access to the service for women with urinary incontinence and pelvic floor dysfunction, by seeking the views of patients, physiotherapists and GPs, and identify changes in referral patterns	523 (47 completed an online questionnaire)	Urinary incontinence and pelvic floor problems (including pelvic organ prolapse and pelvic floor weakness)	Demand/waiting times, referral source, attendance rates by referral source, self-referral rates by age group, attendance by ethnicity, health status at discharge, EuroQOL (EQ-5D-5L) questionnaire, incontinence impact questionnaire shortform, pelvic organ prolapse symptom score, physiotherapist’s feedback	1. Patient completes the self-referral form and is then contacted by the service 2. GP referral to the service 3. GP prompts the patient to complete a self-referral form 4. Other healthcare professionals refer the patient 5. Other healthcare professionals prompts the patient to complete a self-referral form
Howard et al. 2018	Australia	Post-inception audit	To document the patient journey and clinical outcomes of women attending a pelvic health clinic led by physiotherapists	105	Urinary frequency, urgency, and/or incontinence, and/or pelvic organ prolapse	Number of days from referral to commencement of clinical care, number of days from initial assessment to discharge, occasions of service, discharge information, referrals to other health professionals, referrals for investigations, use of pessary device, discussion with medical consultant, adverse events, patient satisfaction, Australian Female Pelvic Floor Questionnaire, Assessment of Quality of Life-6D, Global Rating of Change Scale, Health Professional Satisfaction Survey	Referral from GP to urogynaecology clinic. Those patients prioritised as category 3 and with conditions responsive to physiotherapy interventions were eligible for a physiotherapy-led pelvic health clinic
Luebke et al. 2024	The United States of America	Retrospective clinical audit	To better characterise urinary incontinence treatment patterns in primary care, the authors examined prescriptions and referrals to pelvic floor physical therapy and specialist physicians within a large health system	4,382	Urinary incontinence, overactive bladder and urgency	Type of incontinence, cohort demographics, referral rates to pelvic floor physical therapy or specialist, rates of completed first appointment, likelihood and type of urinary incontinence medication prescriptions	Referral from primary care provider to pelvic floor physical therapy
Van Gerwen et al. 2009	The Netherlands	National survey	To provide insight into the initial management (referral to physiotherapist or specialists and prescription of incontinence pads or medication) of newly diagnosed patients with urinary incontinence in general practice	631 (519 women, 112 men)	Urinary incontinence	Prescription of medication for urinary incontinence, referrals to gynaecologist, urologist or physiotherapist, registered episode of care for urinary incontinence with the GP, patient and general practitioner characteristics	GPs referred patients with urinary incontinence to physiotherapy

## Declarations

### Conference Presentations

Abstract presentation by Divya Subramaniam at the National Conference on Incontinence 2025, Grand Chancellor Hotel, Hobart on 8 May 2025.

## References

[1] Lawrence JM, Lukacz ES, Nager CW, Hsu J-WY, Luber KM. Prevalence and co-occurrence of pelvic floor disorders in community-dwelling women. Obstet Gynecol. 2008. 10.1097/AOG.0b013e3181660c1b 18310371

[2] Cardozo L, Rovner E, Wagg A, Wein A, Abrams P. Incontinence 7th edition. Bristol, UK: ICI-ICS. Int Continence Soc; 2023.

[3] Grimes WR, Stratton M. Pelvic floor dysfunction. StatPearls Publishing, Treasure Island (FL); 2025. https://europepmc.org/article/nbk/nbk559246 32644672

[4] Tim S, Mazur-Bialy AI. The most common functional disorders and factors affecting female pelvic floor. Life. 2021;11(12):1397. 10.3390/life11121397. PMC8704638 34947928

[5] Da Costa SH, Pires LMT, Vieira GC, Castro EAB, Moura EA, Engelmann J, et al. Prevalence of pelvic floor disorders and the associated quality of life among institutionalized and noninstitutionalized elderly women: a cross-sectional study. Current Urol. 2024;17(3):184–7. 10.1097/CU9.0000000000000138 PMC10337817 37448617

[6] Deloitte Access Economics. The economic impact of incontinence in Australia: Continence Foundation of Australia. 2011. Accessed August 28, 2024. https://continence.my.salesforce.com/sfc/p/#A0000000KUc9/a/0f000000Hbwi/9EIyXwIgDG7.s2n1CWOjeC_ZqiBkvcyjCmSmoD1j0mc

[7] Hughes AK, Rostant OS, Pelon S. Sexual problems among older women by age and race. J Womens Health. 2015;24(8):663–9. https://www.liebertpub.com/doi/10.1089/jwh.2014.5010 10.1089/jwh.2014.5010 PMC4545296 26061291

[8] Robinson D, Prodigalidad LT, Chan S, Serati M, Lozo S, Lowder J, et al. International urogynaecology consultation chapter 1 committee 4: patients’ perception of disease burden of pelvic organ prolapse. Int Urogynecol J. 2022;33(2):189–210. 10.1007/s00192-021-04997-3. 34977951

[9] Demir N, Yuruyen M, Atay K, Yavuzer H, Hatemi I, Doventas A, et al. Prevalence of fecal incontinence and associated risk factors in elderly outpatients: a cross-sectional study. Aging Clin Exp Res. 2017;29(6):1165–71. 10.1007/s40520-017-0723-x. 28120284

[10] John G, Gerstel E, Jung M, Dällenbach P, Faltin D, Petoud V, et al. Urinary incontinence as a marker of higher mortality in patients receiving home care services. BJU Int. 2014;113(1):113–9. 10.1111/bju.12359. 24053316

[11] Deloitte Access Economics. Economic cost of incontinence in Australia: Continence Foundation of Australia. 2024. Accessed August 28, 2024. https://drive.google.com/file/d/1nYrPDcGF8S4qfiySGofuFcSyAShWs1Is/view

[12] NICE. Pelvic floor dysfunction: prevention and non surgical management: NICE guideline. NG210. 2021. https://www.nice.org.uk/guidance/ng21 35438877

[13] NICE. Urinary incontinence and pelvic organ prolapse in women: management. BJU Int. 2019;123(5):777–803. 10.1111/bju.14763 31008559

[14] Dumoulin C, Booth J, Cacciari L, Campbell P, Hagen S, Homsi Jorge C, et al. Adult conservative management. (2023). In Abrams P, Cardozo L, Wagg A, Wein AJ, Rovner E (eds.). Incontinence: 7th International consultation on incontinence. Int Continence Soc. 2023:7:795–1038. https://catalogue.curtin.edu.au/view/delivery/61CUR_INST/12233118400001951

[15] Tinetti A, Weir N, Tangyotkajohn U, Jacques A, Thompson J, Briffa K. Help-seeking behaviour for pelvic floor dysfunction in women over 55: drivers and barriers. Int Urogynecol J. 2018;29(11):1645–53. 10.1007/s00192-018-3618-2. 29552740

[16] Tricco AC, Lillie E, Zarin W, O’Brien KK, Coquhoun H, Levac D, et al. PRISMA extension for coping reviews (PRISMA-ScR): checklist and explanation. Ann Intern Med. 2018;169(7):467–73. 10.7326/m18-0850. 30178033

[17] Association of Chartered Physiotherapists in Women’s Health. Information paper project to evaluate patient self-referral to women’s health physiotherapy pilot sites. Charted Soc of Physiotherapy; 2013. PD105. Accessed June 23, 2024 https://www.csp.org.uk/system/files/pd105_womens_health_self_referral_pilot_project_report_2013_0.pdf

[18] Luebke MC, Davidson ERW, Crotty BH, Fergestrom N, O’Connor RC, Schmitt E, et al. Referral and prescription patterns for female patients with urinary incontinence. Urogynecol. 2024;30(5):489–97. 10.1097/SPV.0000000000001423 PMC11002977 37881958

[19] Van Gerwen MA, Schellevis FG, Lagro-Janssen AL. Management of urinary incontinence in general practice: data from the second dutch national survey. J Eval in Clin Pract. 2009;15(2):341–5. 10.1111/j.1365-2753.2008.01012.x 19335495

[20] Howard Z, Jackman A, Bongers M, Corcoran K, Nucifora J, Weir K, et al. Outcomes of a physiotherapy-led pelvic health clinic. Aust & N.Z Cont J. 2018;24(2):43–50. https://www.researchgate.net/publication/326696270_Outcomes_of_a_physiotherapy-led_pelvic_health_clinic

[21] Morrill M, Lukacz ES, Lawrence JM, Nager CW, Contreras R, Luber KM. Seeking healthcare for pelvic floor disorders: a population-based study. Am J Obstet Gynecol. 2007;197(1):86.e81-86. https://pubmed.ncbi.nlm.nih.gov/17618770/10.1016/j.ajog.2007.02.051 17618770

[22] Wang T, Tan JB, Liu XL, Zhao I. Barriers and enablers to implementing clinical practice guidelines in primary care: an overview of systematic reviews. BMJ Open. 2023;13(1):e062158. 10.1136/bmjopen-2022-062158 PMC9827241 36609329

[23] Australian Communications and Media Authority (ACMA). Communications and media in Australia: The digital lives of older Australians. ACMA; 2021. Accessed August 20, 2024. https://www.acma.gov.au/sites/default/files/2021-05/The%20digital%20lives%20of%20older%20Australians.pdf

[24] Campbell K, Drummond A. Management practices for urinary incontinence in women in the primary care setting: health care professionals’ perspective. J POGP. 2021;128:27–38.

[25] Sharma S, Jensen MP, Moseley GL, Abbott JH. Results of a feasibility randomised clinical trial on pain education for low back pain in Nepal: the pain education in Nepal-low back pain (PEN-LBP) feasibility trial. BMJ Open. 2019;9(3):e026874. 10.1136/bmjopen-2018-026874 PMC6475174 30918037

