# Supplementary material for: What is the Journey to Physiotherapy Care for Women over 60 Years with Pelvic Floor Dysfunction: Scoping Review
Source: Int Urogynecol J. 2026 Feb 25;37(9):2743–53. doi: 10.1007/s00192-026-06544-4 (PMC13649927; doi:10.1007/s00192-026-06544-4)
Supplement: Supplementary file 1 [file 192_2026_6544_MOESM1_ESM.pdf]

Supplementary File 1. Search Strategy

1. Proquest

#1 (Anywhere except full text) “women over 60 year*” OR “Female over 60 year*” OR “Older age” OR “Post-menopausal” OR Menopause OR “Older population” OR “Aging women” OR “woman over 60 year*” OR “Aging woman”

#2 (Anywhere except full text) “Pelvic floor dysfunction” OR Incontinence OR “Urinary incontinence” OR “Fecal incontinence” OR “Anal incontinence” OR “Bladder dysfunction” OR “Bowel dysfunction” OR “Sexual dysfunction” OR “Pelvic organ prolapse” OR Dyspareunia OR Vulvodynia OR “Pelvic pain” OR “Pelvic floor disorder” OR “Pelvic floor disease”

#3 (Anywhere except full text) “Pelvic health physiotherapy” OR “Pelvic health physical therapy” OR “Physiotherapy” OR “Physical therapy”

#4 (anywhere except full text) Journey OR Referral OR Pathway

#5 (anywhere except full text) “women over 60” AND incontinence AND referral AND Physiotherapy

#1 AND #2 AND #3 AND #4

2. Scopus

#1 (Article, title, abstract keywords) “women over 60 year*” OR “Female over 60 year*” OR “Older age” OR “Post-menopausal” OR Menopause OR “Older population” OR “Aging women” OR “woman over 60 year*” OR “Aging woman”

#2 (Article, title, abstract keywords) “Pelvic floor dysfunction” OR Incontinence OR “Urinary incontinence” OR “Fecal incontinence” OR “Anal incontinence” OR “Bladder dysfunction” OR “Bowel dysfunction” OR “Sexual dysfunction” OR “Pelvic organ prolapse” OR Dyspareunia OR Vulvodynia OR “Pelvic pain” OR “Pelvic floor disorder” OR “Pelvic floor disease”

#3 (Article, title, abstract keywords) “Pelvic health physiotherapy” OR “Pelvic health physical therapy” OR “Physiotherapy” OR “Physical therapy”

#4 (Article, title, abstract keywords) Journey OR Referral OR Pathway

#5 (Title, abstract, keywords) “women over 60” AND incontinence AND referral OR Physiotherapy

#1 AND #2 AND #3 AND #4

3. Web of Science

#1 (Topic) “women over 60 year*” OR “Female over 60 year*” OR “Older age” OR “Post-menopausal” OR Menopause OR “Older population” OR “Aging women” OR “woman over 60 year*” OR “Aging woman”

#2 (Topic) “Pelvic floor dysfunction” OR Incontinence OR “Urinary incontinence” OR “Fecal incontinence” OR “Anal incontinence” OR “Bladder dysfunction” OR “Bowel dysfunction” OR “Sexual dysfunction” OR “Pelvic organ prolapse” OR Dyspareunia OR Vulvodynia OR “Pelvic pain” OR “Pelvic floor disorder” OR “Pelvic floor disease”

#3 (Topic) “Pelvic health physiotherapy” OR “Pelvic health physical therapy” OR “Physiotherapy” OR “Physical therapy”

#4 (Topic) Journey OR Referral OR Pathway

#5 (abstract) pathway AND physiotherapy AND women AND pelvic organ prolapse

#6 (abstract) pathway AND physiotherapy AND women AND incontinence

#1 AND #2 AND #3 AND #4

4. Pub Med

#1 (Title/abstract) “women over 60 year*” OR “Female over 60 year*” OR “Older age” OR “Post-menopausal” OR Menopause OR “Older population” OR “Aging women” OR “woman over 60 year*” OR “Aging woman”

#2 (Title/Abstract) “Pelvic floor dysfunction” OR Incontinence OR “Urinary incontinence” OR “Fecal incontinence” OR “Anal incontinence” OR “Bladder dysfunction” OR “Bowel dysfunction” OR “Sexual dysfunction” OR “Pelvic organ prolapse” OR Dyspareunia OR Vulvodynia OR “Pelvic pain” OR “Pelvic floor disorder” OR “Pelvic floor disease”

#3 (Title/Abstract) “Pelvic health physiotherapy” OR “Pelvic health physical therapy” OR “Physiotherapy” OR “Physical therapy”

#4 (Title/Abstract) Journey OR Referral OR Pathway

#5 (All text) “women over 60 year*” OR “Female over 60 year*” OR “Older age” OR “Post-menopausal” OR Menopause OR “Older population” OR “Aging women” OR “woman over 60 year*” OR “Aging woman”

#6 (All text) “Pelvic floor dysfunction” OR Incontinence OR “Urinary incontinence” OR “Fecal incontinence” OR “Anal incontinence” OR “Bladder dysfunction” OR “Bowel dysfunction” OR “Sexual dysfunction” OR “Pelvic organ prolapse” OR Dyspareunia OR Vulvodynia OR “Pelvic pain” OR “Pelvic floor disorder” OR “Pelvic floor disease”

#7 (All text) “Pelvic health physiotherapy” OR “Pelvic health physical therapy” OR “Physiotherapy” OR “Physical therapy”

#8 (All text) Journey OR Referral OR Pathway

#1 AND #2 AND #3 AND #4

#5 AND #6 AND #7 AND #8

5. CINAHL

#1 (Abstract) “women over 60 year*” OR “Female over 60 year*” OR “Older age” OR “Post-menopausal” OR Menopause OR “Older population” OR “Aging women” OR “woman over 60 year*” OR “Aging woman”

2 (Abstract) “Pelvic floor dysfunction” OR Incontinence OR “Urinary incontinence” OR “Fecal incontinence” OR “Anal incontinence” OR “Bladder dysfunction” OR “Bowel dysfunction” OR “Sexual dysfunction” OR “Pelvic organ prolapse” OR Dyspareunia OR Vulvodynia OR “Pelvic pain” OR “Pelvic floor disorder” OR “Pelvic floor disease”

#3 (Abstract) “Pelvic health physiotherapy” OR “Pelvic health physical therapy” OR “Physiotherapy” OR “Physical therapy”

#4 (Abstract) Journey OR Referral OR Pathway

#5 (All text) “women over 60 year*” OR “Female over 60 year*” OR “Older age” OR “Post-menopausal” OR Menopause OR “Older population” OR “Aging women” OR “woman over 60 year*” OR “Aging woman”

#6 (Subject) “Pelvic health physiotherapy” OR “Pelvic health physical therapy” OR “Physiotherapy” OR “Physical therapy”

#7 (All text) Journey OR Referral OR Pathway

#8 (Title) women AND Referral and “pelvic health” OR incontinence AND physiotherapy OR physical therapy AND referral

#1 AND #2 AND #3 AND #4

#2 AND #3 AND #5 AND #7

#5 AND #2 AND #6 AND #7

6. Pedro

#1 (Simple search) Journey to pelvic floor physiotherapy

#2 (Simple search) referral to pelvic health physiotherapy

#3 (Simple search) women with pelvic floor dysfunction to pelvic health physiotherapy

#4 (Advanced search) wom* pathway, Problem: incontinence Subdiscipline: gerontology

#5 (Advanced search) referral, Problem: incontinence

7. Google Scholar

#1 referral AND “women over 60 years” AND "pelvic health physiotherapy" AND "pelvic floor dysfunction"

#2 referral AND women over 60 years AND "pelvic health physiotherapy" AND "pelvic floor dysfunction"

#3 the journey of women over 60 to "pelvic health physiotherapy" with "pelvic floor dysfunction"

#4 guidelines for women over 60 to "pelvic health physiotherapy" with "pelvic floor dysfunction"

#5 what is the referral for women with incontinence

#6 physiotherapy referral for post-menopausal women with incontinence

8. Trove

#1 referral to pelvic health physiotherapy

#2 women with pelvic floor dysfunction to pelvic health physiotherapy

#3 referral pathway for women with incontinence to physiotherapy

#4 women AND incontinence AND physiotherapy

9.Google

#1 referral for pelvic health physiotherapy for women over 60 years with pelvic floor dysfunction

#2 journey for pelvic health physiotherapy for women over 60 years with pelvic floor dysfunction

#3 pathway for pelvic health physiotherapy for women over 60 years with pelvic floor dysfunction

#4 guidelines for pelvic health physiotherapy for women over 60 years with pelvic floor dysfunction

#5 “women over 60” AND referral AND pelvic health physio

#6 referral for incontinence AND women over 60 AND physiotherapy

10. DuckDuckGo

#1 how do women over 60 post menopause get to pelvic health physiotherapy

#2 pathway pelvic health physiotherapy menopause women

#3 pathway pelvic health physiotherapy menopause pelvic pain bladder dysfunction bowel dysfunction women

#4 pathway referral pelvic health physiotherapy menopause pelvic pain bladder dysfunction bowel dysfunction women

#5 how do I get to a pelvic health physiotherapist

11. Australian Open Access Repositories

#1 women incontinence

#2 pathway to physiotherapy for women over 60

#3 “women over 60” AND incontinence AND “pathway to physiotherapy”
